# Thousand cankers disease in *Juglans*: Optimizing sampling and identification procedures for the vector *Pityophthorus juglandis*, and the causal agent *Geosmithia morbida*

**DOI:** 10.1016/j.mex.2020.101174

**Published:** 2020-12-03

**Authors:** Salvatore Moricca, Matteo Bracalini, Alessandra Benigno, Luisa Ghelardini, Edson Luiz Furtado, Celso Luis Marino, Tiziana Panzavolta

**Affiliations:** a Department of Agricultural, Food, Environmental and Forestry Science and Technology (DAGRI), Plant Pathology and Entomology section, University of Florence, Italy; b Plant Protection Department, Faculdade de Ciências Agronômicas (FCA), Universidade Estadual Paulista (UNESP), Botucatu, São Paulo, Brazil; c Instituto de Biociências, Departamento de Genética, Universidade Estadual Paulista Júlio de Mesquita Filho (UNESP), Botucatu, São Paulo, Brazil

**Keywords:** Bark beetle, Ascomycete fungus, Quarantine organisms, Funnel traps, Fungus isolation, Macro-, micro-morphological features, Molecular identification

## Abstract

Lindgren funnel traps were used to monitor *Pityophthorus juglandis* occurrence*.* Traps were placed directly on walnut trees, with the top tied to one of the lower branches (about 2m high). An 8-funnel model was used instead of a 4-funnel trap, with the specific pheromone bait positioned between the fourth and the fifth funnel. Traps were customized with a 5mm metal mesh which was placed inside the bottom funnel so that debris (mainly foliage) and larger non-target insects would not end up inside the collecting jar. *Geosmithia morbida* was isolated from beetle adults, larvae and necrotic woody tissue around beetle galleries. Contaminant-free colonies were subcultured in purity and identified by: a) colony phenotyping [morphology, texture and pigmentation; margin type (regular/irregular; lobed/non-lobed); mycelium compactness; surface bumpiness; growth/temperature relationships]; b) micromorphology: type, morphology and ontogeny of conidiophores, metulae and phialides; conidiogenesis; shape, dimension and pigmentation of conidia; c) DNA fingerprinting.

• Our protocol was customized to prevent traps from swinging in the wind and to optimize beetle catches by transversely fixing the bottom of funnel traps to the tree trunk with wooden shafts for stability.
• To enhance fungus isolation in purity, a semi-selective Potato Dextrose Agar (PDA) medium, enriched with the antibiotics Ampicillin (Policillin-N) and Rifampicin (Rifamycin), was devised to prevent contamination by Gram-positive and Gram-negative bacteria and by mycobacteria.

## Graphical abstract

**Figure:**
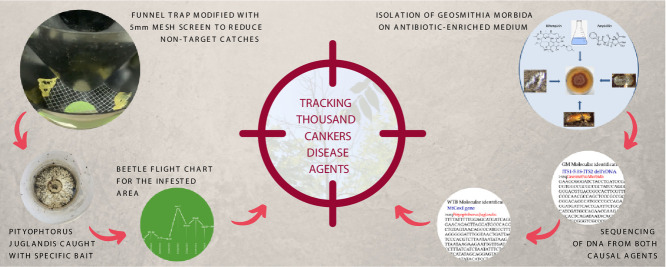

Specifications Table

**Table:** 

Subject Area:	
More specific subject area:	Plant pathology and Entomology
Method name:	Custom tools for WTB and GM surveillance and detection
Name and reference of original method:	Seybold, S.J., Dallara, P.L, Hishinuma, S.M., Flint, M.L., 2013. Detecting and identifying the walnut twig beetle: Monitoring guidelines for the invasive vector of thousand cankers disease of walnut. UC IPM Program, University of California Agriculture and Natural Resources, 13 pp. http://ipm.ucanr.edu/thousandcankers. Erwin D.C., Ribeiro O.K., 1996. *Phytophthora* diseases worldwide. American Phytopathological Society Press, St. Paul, MN.
Resource availability:	*https://www.amazon.com/ULTECHNOVO-Strainer-Funnel-Micron-Vehicle/dp/B07ZZ4YB8Q* *https://www.amazon.com/Propylene-Glycol-Quart-Kosher-Pure/dp/B005F5OJG6/ref=sr_1_2?crid=3J8042HHXQXX&dchild=1&keywords=propylene+glycol&qid=1586162315&sprefix=propylen%2Celectronics%2C257&sr=8-2* https://www.sigmaaldrich.com/catalog/product/roche/roamp?lang=it&region=IT https://www.liofilchem.com/prodotti/prodotti-microbiologia-industriale-clinica/categoria-prodotti.html?classe=018 https://www.gimapackaging.com/it/189-cellophane-trasparente Zeiss light microscope (ZEISS, Jena, West Germany) Leica Wild M8 stereoscope (Leica Microsystems Heerbrugg GmbH, Heerbrugg, Switzerland) GenElute plant Genomic DNA Miniprep extraction kit (Sigma Aldrich, St. Louis, Missouri, USA) Potato dextrose Agar (Liofilchem Srl, Roseto degli Abruzzi, Teramo, Italy) Ampicillin (Sigma Aldrich, St. Louis, Missouri, USA) Rifampicin (Sigma Aldrich, St. Louis, Missouri, USA) Cellophane dishes (Gima Packaging srl Nardò, Lecce, Italy)

## Method details

The present paper reports a customized methodology for sampling and identifying the beetle-pathogen complex responsible for the Thousand Cankers Disease (TCD) in black walnut (*Juglans nigra* L.) [1]. This disease complex includes the recently-described fungus *Geosmithia morbida* Kolařik (GM) (Ascomycota, Hypocreales, Bionectriaceae) and its vector *Pityophtorus juglandis* Blackman (Coleoptera, Curculionidae, Scolytinae), a bark beetle also known as the walnut twig beetle (WTB). The procedure described here was implemented to track the occurrence of the beetle and the fungus for the first time in Tuscany, central Italy, and to identify unequivocally both causal agents [2,3].

## Field

### Study site

Experiments were carried out in an artificial black walnut plantation, with more than half of the trees in an advanced state of decline and exhibiting symptoms typical of TCD: generalized dieback with canopy transparency; leaf yellowing and withering; flag-like brown leaf wilting; and, most importantly, WTB galleries (identified by size-compatible emergence holes) and GM cankers (necrotized areas around holes over the branches and the stems). The area (43°46′ N, 11°24′ E, about 115 m above sea level) has a 1.5 Ha extension, with 281 trees, 90% black walnuts (253 trees) and 10% English walnuts (28 trees). The English walnut trees were apparently asymptomatic and unaffected by TCD. Trees were about 25 years old with diameters (at 1.30 m from the base) ranging between 15 and 35 cm.

### Sampling

**Fig. 1:**
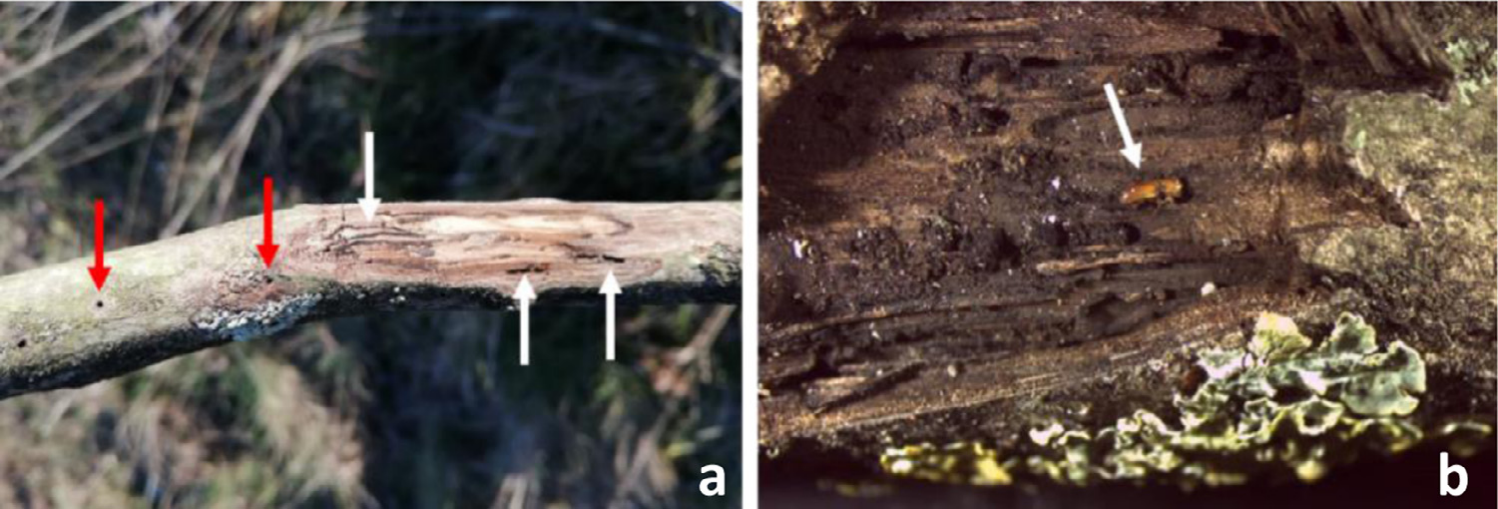
Samples from attacked walnut trees: a) small black walnut branch with walnut twig beetle (WTB) emergence holes (red arrows) and galleries (white arrows); b) WTB adult (arrow) inside one of its galleries.
- GM: to sample the fungus, the walnut plantation was divided into four plots, roughly same-sized, based on the four compass points. From each plot, small (roughly 30–50 cm-long), asymptomatic branches of walnut trees were collected, as well as same-dimension branches with visible beetle entry/exit holes (in some galleries the typical whitish mycelium was visible). The material taken from each tree was placed in polyethylene bags; each tree from which the sample had been taken was labelled, and the same number/code was marked on the sampling bag. This material was then taken to the laboratory for mycological examination.

- WTB: random collecting of dead branches with a long-reach pruner, looking for WTB infested tissues. Samples were checked for the presence of WTB emergence holes on the bark, as well as for galleries under the bark by peeling a small portion of the branch with a knife (Fig. 1). 30 sections of infested branches (40 cm long) were placed in a plastic bag and transferred to the laboratory for further examination.

### WTB trap monitoring

**Fig. 2:**
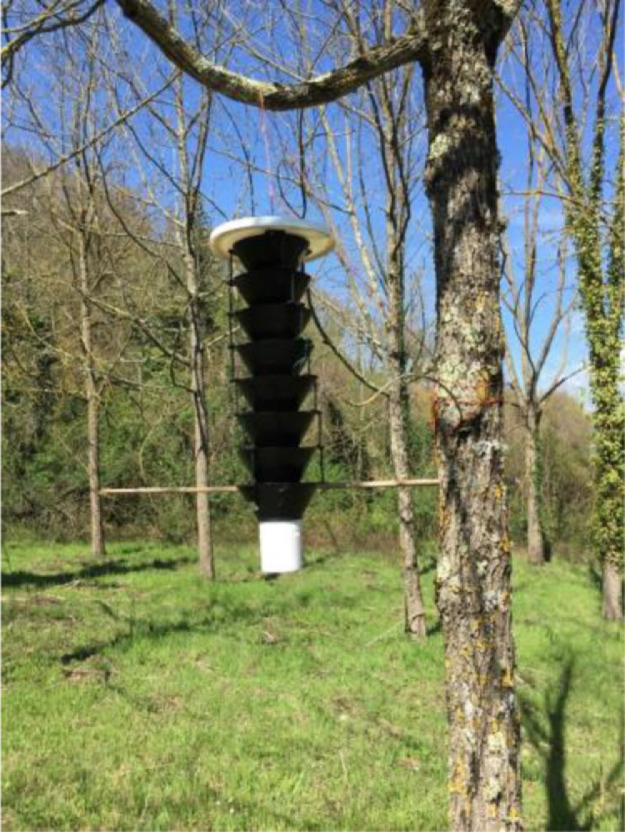
Funnel trap placed on a lower branch of a black walnut by fixing its lower funnel to the tree trunk with a horizontal shaft to reduce swaying from the wind.

Two black plastic funnel traps (eight-unit Lindgren multiple funnel traps - Contech Enterprises Inc., Victoria, BC, Canada) baited with a WTB-specific lure (Alpha Scents Inc., Portland, OR) were placed on the symptomatic black walnut plantation during the first week of April 2018. The top of the trap was hung on low branches (about two meters high) and a transverse shaft was tied between the lower part of the trap and the tree trunk so that the trap would remain in place even in high wind conditions (Fig. 2).

To prevent non-target catches, as well as collection of debris, a 5-mm, metal wire mesh screen was applied low in the bottom funnel of each trap as in Martin *et al*. [4]. The wire mesh was glued inside the funnel by applying four diametrically opposed points of hot glue. The WTB lure consisted of a dispenser which, once peeled open, released a synthetic aggregation pheromone specific to WTB. Dispensers were tied inside the middle funnel and replaced monthly according to manufacturer's instructions. According to Seybold *et al*.’s method [5], the collecting jar was filled with a solution of 30% propylene glycol to allow the prompt killing of catches and their preservation between trap checks; the solution was obtained by adding seven parts of distilled water to pure food-grade propylene glycol.

Traps were inspected every 15 days. During inspection, all debris, mainly fallen foliage, was shaken and then removed from the bottom funnel before detaching the collecting jar. This allowed any remaining beetle to fall past the mesh and into the collecting jar. Then, the jar was unscrewed, and its contents poured into a bottle through a cone paper filter with a fine nylon mesh tip (the kind used for straining car paint or car oil), thus quickly discarding the propylene glycol without losing any beetle specimens. The process was repeated two more times, to rinse the jar thoroughly and to ensure all the WTB specimens were collected, before discarding the propylene glycol. The filter was then folded two times and stored inside a labelled plastic bag, before specimens were transferred to 70% ethanol. The number of WTB specimens captured at each inspection date is reported in Table 1.

**Table 1:** Number of *Pityophthorus juglandis* specimens (males and females) captured in the two funnel traps.

Date	Trap n.1		Trap n.2	
	Females	Males	Females	Males
20/04/2018	10	8	33	18
04/05/2018	18	79	108	97
18/05/2018	31	19	39	34
01/06/2018	25	15	22	26
15/06/2018	333	239	343	218
29/06/2018	60	45	164	249
13/07/2018	108	95	320	239
27/07/2018	87	61	179	153
10/08/2018	109	191	100	88
24/08/2018	182	132	202	171
07/09/2018	102	74	340	348
21/09/2018	65	62	294	199
05/10/2018	36	18	233	184
19/10/2018	53	31	657	450
02/11/2018	73	40	211	133
23/11/2018	5	3	0	1
10/12/2018	0	0	0	1

## Laboratory

### Branch examination

All samples were examined for emergence holes and then their bark was totally removed. All WTB emergence holes and galleries were recorded and some of them were documented with photographs. The WTB specimen was photographed as well, and collected for further analysis (i.e. fungus isolation).

### Nutrient medium preparation

The Potato Dextrose Agar (PDA) medium (39 g PDA, 5 g of agar L^-1^ deionized water) (Liofilchem Srl, Roseto degli Abruzzi, Teramo, Italy) was used to isolate the fungus. The substrate was previously sterilized at 121°C for 15 min, then left to cool to about 45°C, at which temperature antibiotics were added (when the medium was still liquid, to ensure a uniform distribution of antibiotics, but at a temperature below 50°C, to avoid their degradation by heat). 500 µg/ml^-1^ Ampicillin (Policillin-N) and 10 µg/ml^-1^ Rifampicin (Rifamycin) were added, to prevent the growth of Gram-positive and Gram-negative bacteria and of mycobacteria [6]. The antibiotics had been dissolved in 4 ml of absolute ethanol and added to 300 ml of sterile distilled water before being incorporated into the cooling medium.

### GM isolation

GM isolation was carried out from the beetle (adults and larvae) as well as from the wood. Isolation was also carried out from those galleries showing a dense felt of whitish mycelium, by lightly swiping a sterile needle over these mycelial masses and placing the collected material directly into a Petri dish filled with nutrient medium.

For fungus isolation from the beetle, insects were gently removed from the galleries using sterilized tweezers, then roughly 50 specimens were placed into a storage microtube. Within a few hours, insects were placed on the surface of the PDA medium. Beetle larvae were searched for inside underbark tunnels by digging diagonal tunnels with a sharp knife. The larvae found were gently removed with the tip of a brush and placed on the nutrient medium. Those beetle adults and larvae that were still alive and mobile were individually plated into single Petri dishes. In such cases, to avoid unnecessary waste of nutrient substrate, 6 cm-diameter plastic Petri dishes were used.

For fungus isolation from the wood, symptomatic tissue fragments were surface-sterilized by immersion in 96% ethanol (30 s), 4% NaOCl (4 min), followed by another rinsing in 96% ethanol (30 s). The bark was peeled off and wood samples were aseptically excised with a sterile scalpel from the edge zone between healthy and necrotized tissue around holes. Additional tissue pieces were randomly taken from the inside of galleries. Tissue samples were plated onto 9 cm-diameter plastic Petri dishes, in the number of 5 pieces per dish, with one tissue piece placed in the center and the remaining four pieces at the four cardinal points, at roughly 1 cm from the edge of the dish. Colony initials were visible within 24 h, and they were inspected daily in order to remove promptly those contaminated by unwanted fungal saprophytes (contamination rate did not exceed 10%). After 7 days of incubation at 24°C in darkness, single colonies (average diameter 2–3 cm) were transferred to a fresh, basic PDA medium without antibiotics, being these unnecessary to grow colonies in purity at this stage. Pure colonies were incubated again at 24°C in darkness.

### GM mycological identification

**Fig. 3:**
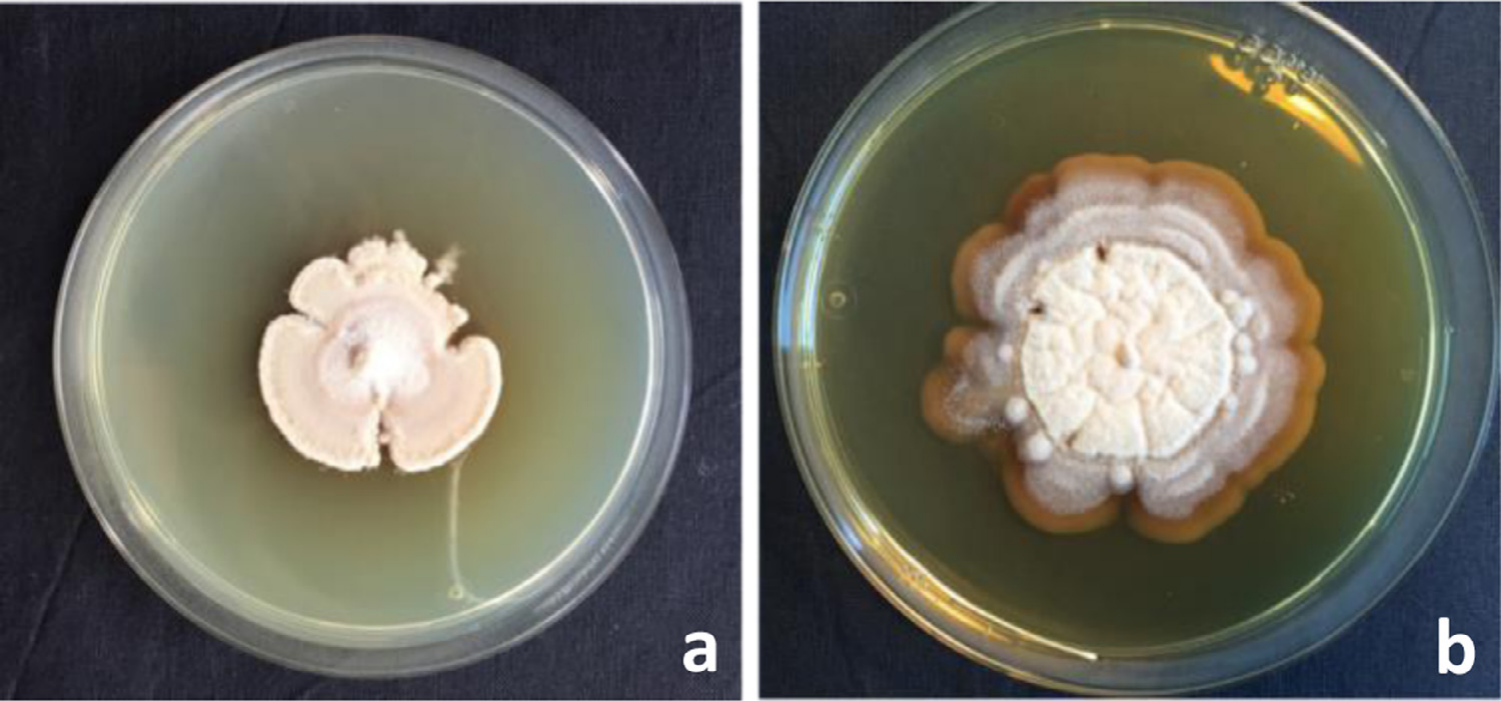
Colonies of *Geosmithia morbida*: a) after 10 days of incubation on PDA medium exhibiting felty, compact texture, widely lobed margin and compact, white mycelium; b) after 15 days of incubation on PDA medium, showing an irregular but clearly defined, brown margin and whitish, short aerial hyphae.

After 10 days of incubation in darkness, when pure subcultures had almost covered the entire surface of the Petri dishes, colony phenotypes were visually determined. Colony phenotypes (Fig. 3), closely resembling the original descriptions of *G. morbida* of Kolarik e*t al.* [1], were individually subjected to microscopic examination. Tufts of mycelium were mounted in lactophenol blue or in lactic acid. Hyphae, conidiophores, metulae, phialides and conidia were examined under a Zeiss light microscope (ZEISS, Jena, West Germany) at x100, x400 and x1000 magnifications.

### WTB identification

**Fig. 4:**
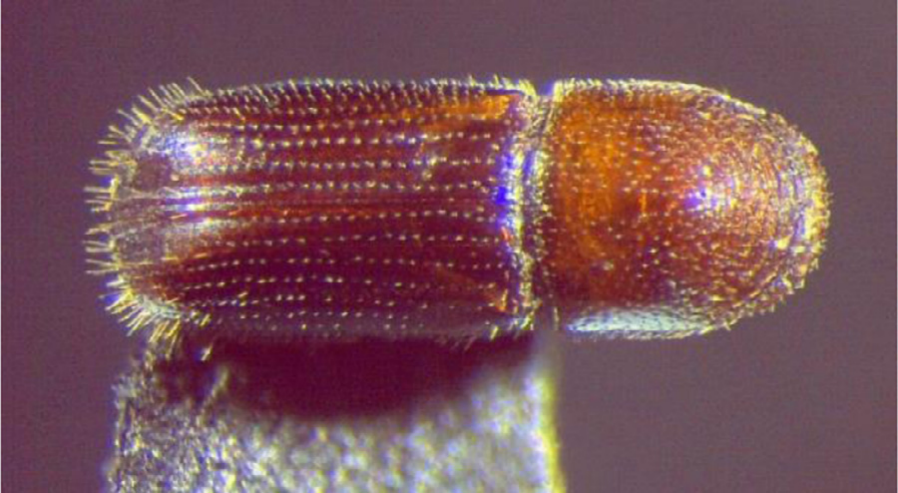
Dorsal view of an adult walnut twig beetle showing the concentric rows of asperities in the anterior half of the pronotum.

Once in the laboratory, all bark beetles were isolated from fine debris as well as other non-target insect catches which were small enough to bypass the wire mesh screen. Then, their identification was initially verified via morphological observation with a Leica Wild M8 stereoscope (Leica Microsystems Heerbrugg GmbH, Heerbrugg, Switzerland) at 100x magnifications [7,8] Specifically, the overall size of the specimens (less than 2 mm) and the concentric rows of the numerous pronotal asperities from the middle to the anterior margin, were among the most important features looked for (Fig. 4). Also, following the taxonomic information provided by the same authors, the sex ratio was measured by identifying male and female WTBs: discriminating traits were the interstriae of the elytral declivity and the pubescence on the frons.

## DNA-based identification

### GM

For DNA extraction, each isolate was inoculated onto 9 cm diameter Petri dishes containing PDA on whose surface a sterile cellophane disc had previously been laid on. Cellophane discs (Gima Packaging srl Nardò, Lecce, Italy) separated the fungal mycelium from the medium, while allowing nutrient exchange and making the colony picking up easier without contaminating the mycelium samples with culture medium. The dishes were incubated at 24°C for 7 days, enough time for the growing mycelium to cover the entire surface of the dish. The mycelium was picked up by using a sterile scalpel, then placed into a sterile Eppendorf tube. Ethanol was then added, to avoid possible contamination and to facilitate the lysis of the mycelium. After 3 days, the ethanol was carefully removed and the DNA was extracted using the GenElute plant Genomic DNA Miniprep extraction kit (Sigma Aldrich, St. Louis, Missouri, USA) according to manufacturer's recommendations and stored at -20°C. The Internal Transcribed Spacer (ITS) region was amplified using the universal primers ITS6 (5′GAAGGTGAAGTCGTAACAAGG-3′) [9] and ITS4 (5′-TCCTCCGCTTATTGATATGC-3′) [10]. The PCR mixing protocol was the same as described above for WTB samples. PCR conditions were an initial denaturation step of 3 min at 95°C, followed by 35 cycles of denaturation for 30 s at 95°C, annealing for 30 s at 55°C and extension for 1 min at 72°C, followed by a final elongation of 5 min at 72°C [11]. Amplicon sizes were checked by electrophoresis in a 1% agarose gel (Sigma Aldrich, St. Louis, Missouri, USA) and then sequenced.

### WTB

Molecular identification of the beetle was performed by extracting DNA from 19.31 mg of dead individuals using disposable 1.5 ml microfuge tubes and sterilized pestles. The homogenized material was then processed using the same GenElute plant Genomic DNA Miniprep protocol used for the fungus (see above), and the resulting DNA was stored at -20°C until usage. The cytochrome oxidase subunit 1 (CO1) mitochondrial gene region was PCR-amplified and sequenced using the universal primers LCO1490F and HCO2198R [12]. Amplification was performed through 35 cycles of 1 min at 95°C, 1 min at 40°C, and 1.5 min at 72°C, followed by a final extension at 72°C for 7 min [12].

### Temperature effects on GM growth and survival

Three isolates of *G. morbida* (ROS1, ROS2 and ROS3) were chosen to test fungus growth rates at different temperatures and to check what the critical upper temperature was (cessation of growth and death). Colonies were grown on sterile Petri dishes containing PDA. Mycelial plugs (3 mm) were picked up from the outermost part of the colony with a sterile scalpel and placed in the center of a Petri dish containing the nutrient medium. Three replicates per each isolate (n = 3) were incubated in the dark at 21, 25, 29, 33, 37 and 41°C and the radial colony growth was recorded at two-day intervals. The fungus grew optimally at 25°C and still showed fair growth at 37°C, whereas it was no longer viable (0 growth) at 41°C (Table 2).

**Table 2:** Raw data for colony radial growth of three *G. morbida* isolates (ROS1, ROS2 and ROS3) at different temperatures. Data were recorded at two-day intervals along two lines intersecting the center of the inoculum at a right angle.

	Day 2	Day 4	Day 6	Day 8	Day 10
21°C					
ROS 1	2.6	4.8	6.9	8.8	11.6
ROS 2	1.4	2.9	4.6	7.1	9
ROS 3	1.1	2.7	4.1	5.1	6
25°C					
ROS 1	3.3	6.1	8.5	11.9	14
ROS 2	3.9	6.5	10.4	13.1	15
ROS 3	4.5	8.1	12.8	15.6	19
29°C					
ROS 1	3	4.9	7	9.8	12
ROS 2	3.2	5.4	7.3	10	13.5
ROS 3	2.9	5.2	7.2	9.9	12.5
33°C					
ROS 1	1.8	3.3	5.6	6.9	9
ROS 2	1.7	3.5	4.6	5.5	7.9
ROS 3	1.7	3.4	4.2	5.4	7.5
37°C					
ROS 1	1.6	2	2.4	2.7	3
ROS 2	1.7	2.2	2.6	3.1	3.5
ROS 3	1.7	2.3	2.9	3.7	4
41°C					
ROS 1	0	0	0	0	0
ROS 2	0	0	0	0	0
ROS 3	0	0	0	0	0

## Conclusions

Both *P. juglandis* and *G. morbida* are quarantined organisms in the EU and, as a consequence, the development of effective protocols for their detection is at this moment of particular importance [13]. Our protocols will aid in tracking the occurrence of both causal agents in disease surveillance campaigns in EU territories. Since Kolařik *et al.* [1] had found that the fungus was still viable at a temperature of 41°C, it was also important to acquire data on the optimum and extreme temperature growth rates of fungal isolates. Our data indicate that *G. morbida* is less thermotolerant than previously reported [1]. As the Tuscan TCD outbreak is - at least for the moment - the southernmost one in Europe, understanding the ability of the pathogen to adapt to the warm climates of southern Europe has epidemiological relevance, with direct implications for the risk TCD poses for the widespread cultivation of walnuts in southern Europe.

Our findings will be of interest to researchers, plant growers and government investigators who are faced with this new and alarming phytosanitary emergency.

## Declaration of Competing Interest

The authors declare that they have no known competing financial interests or personal relationships that could have influenced the work reported in this paper.
